# Heterosubtypic Neutralizing Monoclonal Antibodies Cross-Protective against H5N1 and H1N1 Recovered from Human IgM^+^ Memory B Cells

**DOI:** 10.1371/journal.pone.0003942

**Published:** 2008-12-16

**Authors:** Mark Throsby, Edward van den Brink, Mandy Jongeneelen, Leo L. M. Poon, Philippe Alard, Lisette Cornelissen, Arjen Bakker, Freek Cox, Els van Deventer, Yi Guan, Jindrich Cinatl, Jan ter Meulen, Ignace Lasters, Rita Carsetti, Malik Peiris, John de Kruif, Jaap Goudsmit

**Affiliations:** 1 Crucell Holland BV, Leiden, The Netherlands; 2 Department of Microbiology, The University of Hong Kong, Queen Mary Hospital, Hong Kong Special Administrative Region, People's Republic of China; 3 Algonomics NV, Gent-Zwijnaarde, Belgium; 4 Central Veterinary Institute, Wageningen University, Lelystad, The Netherlands; 5 Institute for Medical Virology, Johann Wolfgang Goethe University, Frankfurt am Main, Germany; 6 Laboratory of Cell Biology, Bambino Gesu Children's Research Hospital, Rome, Italy; New York University School of Medicine, United States of America

## Abstract

**Background:**

The hemagglutinin (HA) glycoprotein is the principal target of protective humoral immune responses to influenza virus infections but such antibody responses only provide efficient protection against a narrow spectrum of HA antigenic variants within a given virus subtype. Avian influenza viruses such as H5N1 are currently panzootic and pose a pandemic threat. These viruses are antigenically diverse and protective strategies need to cross protect against diverse viral clades. Furthermore, there are 16 different HA subtypes and no certainty the next pandemic will be caused by an H5 subtype, thus it is important to develop prophylactic and therapeutic interventions that provide heterosubtypic protection.

**Methods and Findings:**

Here we describe a panel of 13 monoclonal antibodies (mAbs) recovered from combinatorial display libraries that were constructed from human IgM^+^ memory B cells of recent (seasonal) influenza vaccinees. The mAbs have broad heterosubtypic neutralizing activity against antigenically diverse H1, H2, H5, H6, H8 and H9 influenza subtypes. Restriction to variable heavy chain gene IGHV1-69 in the high affinity mAb panel was associated with binding to a conserved hydrophobic pocket in the stem domain of HA. The most potent antibody (CR6261) was protective in mice when given before and after lethal H5N1 or H1N1 challenge.

**Conclusions:**

The human monoclonal CR6261 described in this study could be developed for use as a broad spectrum agent for prophylaxis or treatment of human or avian influenza infections without prior strain characterization. Moreover, the CR6261 epitope could be applied in targeted vaccine strategies or in the design of novel antivirals. Finally our approach of screening the IgM^+^ memory repertoire could be applied to identify conserved and functionally relevant targets on other rapidly evolving pathogens.

## Introduction

Influenza pandemics have historically been associated with high levels of morbidity and mortality. Pandemics return periodically and a new pandemic is now overdue. The most serious pandemic threat in recent times has been posed by the highly pathogenic avian influenza virus (HPAI) H5N1 which emerged in South-East Asia in 1997 [1]. Cumulatively 385 cases have been reported with an overall mortality of 63% [2]. In addition other avian influenza viruses including H2, H6, H7 and H9 subtypes have been reported to either have caused human cases or shown potential to do so, and are also recognised as potential pandemic threats [3]–[6].

Preparedness to confront an influenza pandemic is still a major public health issue. Broad spectrum antivirals, such as the neuraminidase inhibitor oseltamivir, have been stockpiled as a first line defence against rapidly spreading HPAI strains. However the use of oseltamivir in the treatment of H5N1 infections has been associated with the generation of resistant viruses [7], [8] and a sharp increase in the isolation of human H1N1 strains resistant to oseltamivir was recorded in 2008 [9] indicating that other preventative measures are required. Pre-pandemic vaccination has been put forward as a strategy to ameliorate the spread of virus and severity of disease, however all vaccines have the limitation that they protect at best against strains from the same subtype [10]. Thus, an immunological intervention that will be active across the spectrum of potential pandemic subtypes and clades remains an elusive goal for influenza prophylaxis and therapy.

Passive immunization has been anecdotally reported to be effective against H1N1 and H5N1 viruses [11], [12], indicating that immunoglobulins might be effective against infection and disease of the systemic nature seen in the H1N1 1918 influenza pandemic. Based on this, broadly cross-neutralizing monoclonal antibodies (mAbs) against the avian influenza virus H5N1 have been pursued using a variety of approaches [13], [14]. These approaches have concentrated on using convalescent patient material as a source of B cells for screening of antibodies. However it has long been known that the immune response against influenza virus is highly restricted [15], as borne out by a recent cloning study [16], and focused on subtype and strain specific epitopes [17], [18]. Thus to access a more diverse immune repertoire we chose the novel strategy of interrogating the human IgM^+^ memory B cell repertoire. Although this subset of B cells is characterised by CD27 expression and mutated V genes, both tightly linked to the memory B cell phenotype, the origin and role of this subset of B cells is controversial. It has been proposed that circulating B cells with this phenotype are linked to marginal zone B cells and have a primary role in T independent immunity [19], [20], while others argue they are formed as part of an intermediate differentiation step in normal T dependent germinal centre immune responses [21]. Several reports have highlighted a role for IgM in the early stages of protection from experimental influenza virus challenge [22], [23]. Intriguingly this protective role includes influenza virus subtypes to which mice are immunologically naïve [23], [24].

Based on our hypothesis that the IgM^+^ B cell subset contains a diverse repertoire of antibodies against conserved epitopes on pathogens we have applied antibody phage display to search for broadly neutralizing H5N1 mAbs using combinatorial libraries built from B cells isolated from donors recently vaccinated with the seasonal influenza vaccine. Using this approach we have rescued a panel of human antibodies that show an unexpected breadth of influenza subtype neutralization that include H5, H1, H2, H6, H8 and H9 (H2, H5, H6, H9 being identified as high risk pandemic candidates). The binding region of these mAbs has been localised to a conserved region of the HA stem domain. The lead mAb CR6261 showed prophylactic and therapeutic efficacy in mouse models with H5N1 or H1N1 challenge.

## Materials and Methods

### Viruses and recombinant proteins

The reverse genetics reassortants (RG) NIBRG-14 (A/Puerto Rico/8/34 with low pathogenic (LP) HA and NA of A/Vietnam/1194/04; H5N1) and RG-A/Indonesia/5/05 (A/Puerto Rico/8/34 with (LP) HA and NA of A/Indonesia/5/05; H5N1) were grown in MDCK cells by standard viral culture techniques. The wild type H5N1 strain A/HongKong/156/97 was originally obtained from a 3-year-old child suffering from respiratory disease [25]. The virus was passaged twice on MDCK cells. The batch (8.1 log TCID50 /ml) used to infect mice was propagated once in embryonated eggs. A/Vietnam/1194/04 (H5), A/Vietnam/1203/04 (H5), A/Indonesia/5/05 (H5), A/Bar headed goose/Qinghai/5/05 (H5), A/Japanese White Eye/Hong Kong/1038/06 (H5), A/HongKong/483/97 (H5), A/Hong Kong/54/98 (H1), A/Hong Kong/201345/07 (H1), A/Singapore/1/57 (H2), A/WF/Hong Kong/MPU3156/05 (H2), A/WF/Hong Kong/MPA2290/06 (H3), A/WF/Hong Kong/MP2437/04 (H6), A/WF/HongKong/MPB127/05 (H7), A/WF/HongKong/MPD373/07 (H8), A/Duck/Y280/97 (H9) were grow in eggs or MDCK cells by standard viral culture techniques. BPL inactivated NIBRG-14 and A/New Caledonia/20/99 were purchased from NIBSC (UK). To generate mammalian expressed soluble recombinant (r) HA, the coding region for A/Vietnam/1194/04 HA excluding the transmembrane region was synthesized and cloned in a pcDNA-based expression vector containing a myc- and his-tag. PER.C6® cells were transfected with the soluble HA expression constructs and rHA was purified from culture supernatant using HisTrap™ FF Columns (GE Healthcare). rHA, subtype H1 (A/New Caledonia/20/99), subtype H3 (A/Wyoming/3/03), subtype H5 (A/Vietnam/1203/04), subtype H7 (A/Netherlands/219/03), subtype H9 (A/Hong Kong/1073/99) and B/Ohio produced using baculovirus vectors in insect cells was purchased from Protein Sciences Corp., (CT, USA).

### Library construction

IgM^+^ memory B cells (CD24+/CD27+/IgM+) were sorted from peripheral blood mononuclear cells obtained with written consent from ten normal healthy donors by fluorescent activated cell sorting (FACS, Digital Vantage, Becton Dickinson). ScFv phage display libraries were constructed essentially as described [26] using ∼ 1×10^5^ sorted IgM memory B cells from each individual donor (Supplementary Fig. 1). To ensure amplification of the IgM memory B cell VH repertoire, an IgM-specific constant region primer was used for the first amplification. The individual libraries of 10 donors were rescued, amplified using CT helper phages [27] and mixed. The pooled libraries used for phage antibody selections consisted of more than 2×10^8^ individual clones.

**Figure 1:**
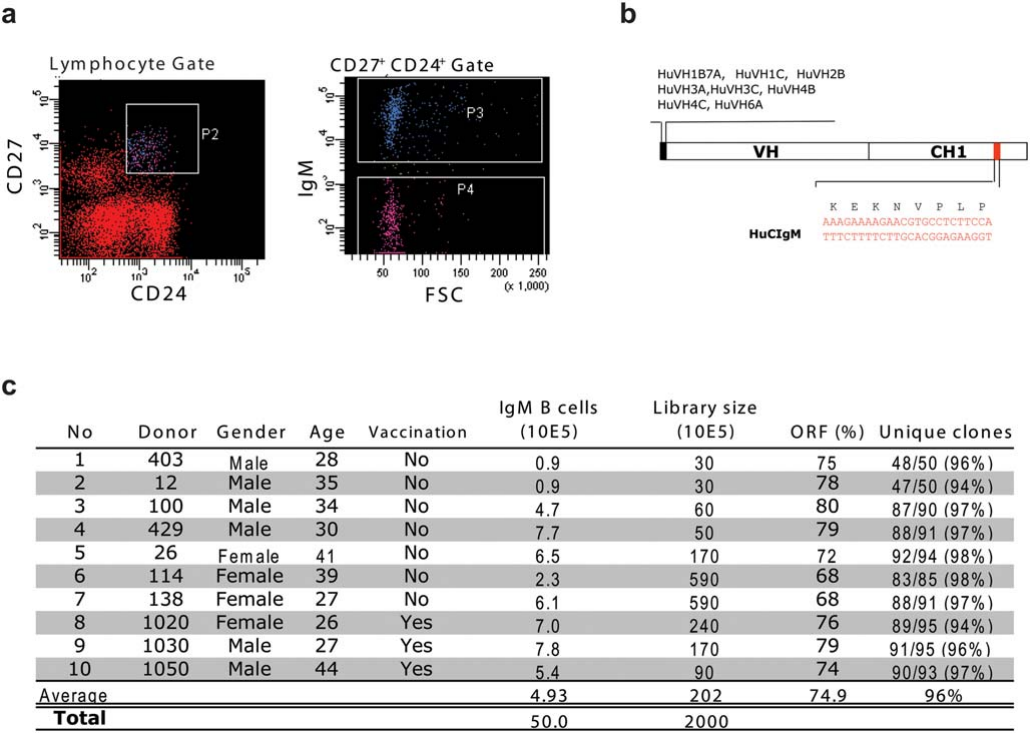
Construction of IgM^+^ memory B cell libraries. a) Donor lymphocytes were isolated by Ficoll-plaque from heparinized blood and stained for the phenotypic markers CD27, CD24 and IgM. CD24^+^ CD27^+^ cells were gated and the IgM^+^ cells within this gate sorted directly into Trizol for RNA extraction. b) RT-PCR was performed using a pool of 5′ oligonucleotide primers that cover all VH gene families and a 3′ oligonucleotide primer that anneals in a region of the CH1 domain of Cμ distinct from other immunoglobulin isotypes. c) Using cDNA generated in this way, 10 individual scFv libraries were constructed as described previously [26]. Donors 1020, 1030 and 1050 had been vaccinated with the Dutch 2005 seasonal influenza vaccine 7 days prior to collection of blood. All libraries demonstrated a high percentage of correct scFv ORF's and diversity based on unique HCDR3 sequence.

### Selection of HA binding clones

Phage panning to soluble rHA and (anti-Myc-captured) soluble rHA (5.0 µg/ml in PBS, pH 7.4) immobilized to MaxisorpTM Immunotubes (Nunc) was performed essentially as described [26], except for the use of 100 mM TEA (Sigma) for phage elution. Alternatively, phage selections were performed in suspension on PER.C6® cells transfected with full length A/Vietnam/1194/04 HA or a consensus HA sequence representing H5N1 strains isolated in Indonesia and China during 2005 as previously described [26]. Rescue and propagation of eluted phages was performed as described. After two rounds of selections, individual scFv-phage antibodies were rescued and tested for binding to rHA and rHA-expressing PER.C6® cells in ELISA and FACS, respectively as previously described [26].

### IgG1 and Fab expression

Fully human IgG1 antibodies were constructed by cloning the heavy (VH) and light (VL) chain variable regions of H5 rHA-specific scFv-phage antibodies into separate vectors for IgG1 heavy and light chain expression. Human IgG1 mAbs were expressed and purified as described previously [28]. Fab expression constructs were generated by recloning the VH region into a vector containing the CH1 region of IgG1 and a myc- and his-tag. HEK293T/17 cells were transfected with the Fab expression construct and the light chain construct and expressed Fabs purified from culture supernatant using HisTrap™ FF Columns (GE Healthcare).

### ELISA binding assays

Microtiter plates (Immuno™ Maxisorp, Nunc) were coated overnight at 4°C with 0.5 µg/ml soluble rHA or BPL inactivated virus then washed three times with PBS containing 0.1% v/v Tween-20 and blocked in PBS containing 2% w/v non-fat dry milk (blocking solution) for 1 hr at RT. For scFv-phage binding assays, scFv-phage antibodies were pre-incubated for 1 hr in an equal volume of PBS containing 4% w/v non-fat dry milk and added to the wells. After 1 hr plates were washed with PBS/0.1% v/v Tween-20 and bound phage antibodies were detected using a peroxidase-conjugated anti-M13 antibody (GE Healthcare). For IgG1 binding assays, anti-HA IgG1 in blocking solution were added to wells and incubated for 1 hour at room temperature, after which bound IgG1 was detected using a peroxidase-conjugated mouse anti-human IgG antibody (Jackson). The reaction was developed with O-phenylenediamine substrate (Sigma FAST OPD; Sigma) and stopped by the addition of 1M H_2_SO_4_. The absorbance was measured at 492 nm. For competition assays, anti-HA IgG1 were incubated as above and after washing incubated with FITC conjugated CR6261 or CR6323 (0.2 ug/ml) for 5 min at RT followed by anti-FITC rabbit IgG HRP; detection was as above.

### FACS binding assays

For scFv-phage binding assays, PEG/NaCl precipitated scFv-phages were mixed with an equal volume of PBS/2% ELK and blocked for 30 minutes on ice. The blocked phages were added to pelleted cells (untransfected PER.C6® and HA-expressing PER.C6® cells) and incubated for one hour on ice. The cells were washed three times with PBS/1% BSA, followed by a 1 minute centrifugation at 300×*g.* Binding of the single chain phage antibodies to the cells was visualized using a biotinylated anti-M13 antibody (Fitzgerald) followed by streptavidin-phycoerythrin conjugate (Caltag). For IgG1 binding assays, 3×10^5^ cells were incubated for 1 h on ice in the presence of serial dilutions of anti-HA IgG1. Cells were washed three times then incubated for 30 min with phycoerythrin-conjugated anti-IgG (Southern Biotech). Stained cells were analyzed using a FACS Calibur with CELLQuest Pro software (Becton Dickinson). To assay for HA2 specific reactivity cells were treated with 10 µg/ml trypsin-EDTA in DMEM for 30 min at RT, washed and incubated for 5 min in acidified PBS (pH 4.9), washed and then incubated for 20 min in the presence of 20 mM DTT at RT. Cells were split at each step and untreated adherent cells were resuspended in 0.05% EDTA. Cells were stained with IgG1 as described above.

### Western Blotting

rHA antigen (0.4 µg/lane) was subjected to SDS-PAGE under reducing conditions alongside the marker Precision Plus (Biorad). Fractionated protein was transferred to Immobulon-P membrane (Millipore), blocked with 4% milk powder and probed with 10 µg/ml anti-H5 IgG1 containing supernatants. Immunoreactivity was detected by chemiluminescence following incubation with a peroxidase-conjugated mouse anti-human IgG antibody (Southern Biotech) and 2 min ECL incubation. Membranes were exposed to film (Hyperfilm, GE Healthcare) and image developed.

### SPR analysis of Fabs

Surface plasmon resonance (SPR) analysis was performed on a BIAcore3000. Sheep anti-NIBRIG-14 serum or FLU mAb IgG was immobilized on a CM5 sensor chip by amine coupling and then used to capture soluble rHA; one channel on each chip was not coated and used as a negative control. Twelve concentrations of Fabs in 2-fold dilutions from 1000 nM down to 0.39 nM were injected at a constant flow rate of 100 µl/min at 25°C. At the end of the injection, running buffer (HBS-EP, pH 7.4) was applied for 770 s, followed by regeneration of the CM5 chip using 5 µl 10 mM NaOH. The experiments were repeated twice. The resulting data were fitted using a standard 1:1 Langmuir model and the dissociation constant KD calculated. BIAcore evaluation software (version 3.2, July 2001) was used throughout.

### Microneutralisation assays

MDCK cells were maintained in minimum essential medium (MEM) supplemented with 10 % fetal calf serum (FCS) and 1% penicillin-streptomycin (PS) at 37°C. On the day of the experiment, MDCK cells in 96-well format were washed twice with PBS and incubated in MEM supplemented with 1% FCS, 1% PS and 1 µg/ml TPCK trypsin (for non-H5 viruses). Two-fold serially diluted purified IgG1 or IgG1 containing supernatant was mixed with an equal volume of viral inoculum, followed by 2 hour incubation at 37°C. After the incubation, the mixture (∼100 TCID_50_) was added to confluent MDCK monolayers. Cells were cultured for 72 hours before the examination of cytopathic effect (CPE). CPE was compared to the positive control (virus-inoculated cells) and negative controls (mock-inoculated cells). The absence of CPE in individual wells was defined as protection. The assay was performed in quadruplicate.

### Hemagglutination inhibition assay

Virus was diluted to 8 HA units and combined with an equal volume of serially diluted IgG1 and incubated for 1 hr at room temperature. An equal volume of 0.5% Turkey red blood cells was added to the wells and incubation continued on a gently rocking plate for 30 min. Button formation was scored as evidence of hemagglutination.

### Generation and Characterizations of Neutralization Resistant variants

RG-A/Indonesia/5/05 was cultured in the presence of purified CR6261 for 10 passages. A control experiment without CR6261 was done in parallel as a reference. Serially diluted (10^−1^ to 10^−6^ folds) viruses were first incubated in the presence of antibody (2.5 µg/ml) for 1 hour at 37°C. This concentration of antibody was previously shown to reduce the H5N1 viral titre by 3 logs (data not shown). The incubated mixture was then absorbed by MDCK cells for 1 hour. Infected cells were washed with PBS twice and replenished with MEM containing 1% FCS and 1% PS, 2.5 µg/ml of CR6261. CPE of these infected cells was monitored 48–72 hrs post-infection. Supernatant from CPE positive wells infected with the lowest virus titre were harvested for a subsequent round of infection. Supernatants from passage 10 were subjected to standard plaque assays and infected cells were overlaid with agar containing CR6261 (2.5 µg/ml). Six individual virus plaques were purified and grown in MDCK cells in the presence of CR6261 (2.5 µg/ml). The HA sequences of these were examined by routine sequencing techniques. Representative mutants were characterized by the neutralization assays as described above.

### Murine lethal challenge models

All experiments were approved prior to commencement by the ethical review committee of the Animal Sciences Group in accordance with Dutch law. Female 7-week-old BALB/c mice were inoculated intranasally on the day indicated with 25 LD_50_ A/HongKong/156/97 (4.5logTCID_50_) or A/WSN/33 (6.6logTCID_50_) or ∼ 10 LD_50_ A/Vietnam/1194/04 (3.7logTCID_50_) and observed daily for clinical signs and weighed. Clinical signs were scored with a scoring system (0 = no clinical signs; 1 = rough coat; 2 = rough coat, less reactive, passive during handling; 3 = rough coat, rolled up, laboured breathing, passive during handling; 4 = rough coat, rolled up, laboured breathing, unresponsive) and recorded. Surviving animals were euthanized and bled on day 21. IgG1 was injected i.p. in a volume of 500 µl or i.v. in the tail vein in a volume of 200 µl.

### Homology Modeling and Docking

Antibody modelling was performed by individually selecting optimal framework and CDR template fragments from crystal structures based on sequence analysis. CDR's were grafted by structural fit, substituted side-chains were optimised by FASTER algorithm [29] and conjugate gradient minimization. The antibody model was then manually docked on the HA crystal structure using the Brugel software.

### PCR amplification of neutralizing mAb VH genes from IgM^+^ memory B cell library cDNA

Library cDNA (200ng) was amplified with the following primer sets sc06261_Fw (5′AGGCCCCTTCCGCAGCTATGCTAT) and sc06261_Rv_v3(5′TTTCGCGCACCTGGTACCCCATATG)

CR6323_L (5′AGGCACCTTCTCCAGCTATG) and CR6323_R (5′GGGGAGGTATGCAGGGTAAT)

CR6325_L (5′GGAGGCACCTTCAGCTTCTA) and CR6325_R (5′GTAGTAGATACCCTTATCACCCTCTC)

CR6329_L (5′GGAGGCATCTTCAGAAGCAA) and CR6329_R (5′CAAAGTAGTTGCGTGTGGTGT)

PCR reactions were performed using PWO polymerase (Roche) and products loaded on 1,5% AGAROSE-TAE gel and detected with SybrSafe (Invitrogen). PCR products with the expected amplicon size were extracted from gel and purified (Zymoclean Gel DNA recovery kit, Baseclear) and cloned into pCR4-TOPO (Invitrogen). Multiple >10 clones were sequenced using standard techniques with the primers M13_Fw and M13_rev.

## Results

### Isolation and characterization of H5N1 specific mAbs

**Figure 2:**
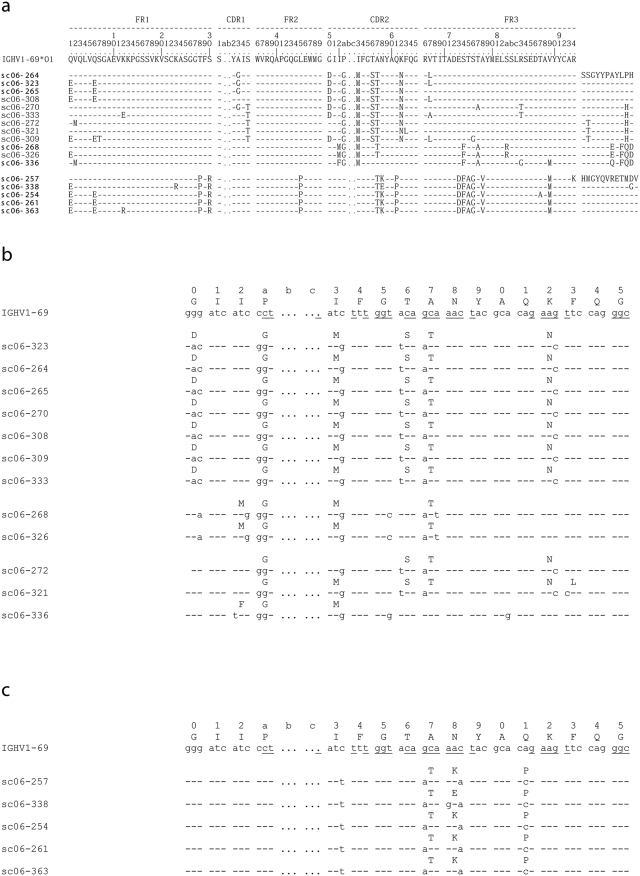
Sequence analysis of VH genes from selected anti-H5 HA scFv. (a) Alignment of VH amino acid sequences from scFv containing the same V(D)J rearrangement as CR6323 or CR6261 with the germline IGHV1-69. Note that although multiple mutations are present in the CDR1 and CDR2 loops Phe at position 54 is conserved (Kabat numbering). scFv that were neutralizing in an IgG1 format are shown in bold (b) DNA alignment and amino acids for the HCDR2 of (b) CR6323 and (c) CR6261 with related mAbs. Note the identical codon usage and conservation of silent mutations suggesting a clonal origin.

Ten individual phage antibody libraries were constructed from sorted IgM^+^ memory B cells of three donors vaccinated with a seasonal influenza vaccine and seven unvaccinated donors (Fig. 1). The libraries were pooled and then used for panning against soluble or surface expressed forms of H5 recombinant (r) hemagglutinin (HA). A total of 223 H5 rHA-specific scFv-phages were isolated. Sequence analysis identified 91 distinct VH genes of which 43 could be traced to a unique V(D)J recombination event that included VH gene families 1,3,4 and 5 (Table 1). All VH genes showed evidence of somatic mutation with an average frequency of 5.3/100 bp, consistent with a previous analysis in this B cell compartment [20]. We also noted that many of the mAbs grouped in terms of V(D)J rearrangement showed signs of clonal expansion based on conserved codon usage (Fig. 2).

**Table 1:** Sequence Characteristics of H5N1 specific scFv

No	ID	Clones	IGHV	IGHD/ frame	IGHJ	VH Mutations/100bp
1	6364	1	1-02	1-01 / 1	4	3.8
2	6366	1	1-02	5-05 / 3	4	4.2
3	6347	2	1-02	7-27 / 3	3	2.1
4	6141	11	1-18	6-06 / 1	6	7.6
5	6365	1	1-69	1-26 / 3	3	0.7
6	6269	8	1-69	1-26 / 3	1	5.9
7	6325	1	1-69	1-26 / 3	6	4.5
8	6334	1	1-69	2/OR15-2a / 1	6	10.8
9	6261	42	1-69	2-02 / 1	6	6.9
10	6332	1	1-69	2-15 / 2	6	8.0
11	6327	1	1-69	3-03 / 2	4	5.2
12	6339	1	1-69	3-03 / 2	4	5.9
13	6344	1	1-69	3-10 / 2	4	6.6
14	6342	1	1-69	3-10 / 2	5	19.1
15	6323	18	1-69	3-22 / 2	1	5.9
16	6343	1	1-69	3-22 / 2	4	6.3
17	6331	1	1-69	3-22 / 2	5	2.8
18	6262	6	1-69	4-04 / 2	6	9.4
19	6329	1	1-69	6-25 / 1	4	9.4
20	6143	1	3-07	3-16 / 3	1	4.2
21	6151	3	3-09	5-05 / 3	4	2.8
22	6307	1	3-21	1-26 / 3	4	1.4
23	5111	31	3-21	2-08 / 2	4	3.8
24	6295	2	3-21	3-10 / 2	4	0.7
25	6302	1	3-23	1-26 / 3	4	6.3
26	6134	1	3-23	3-03 / 2	5	6.3
27	6139	7	3-23	3-10 / 2	4	5.6
28	6144	9	3-23	4-17 / 3	4	3.8
29	6301	1	3-23	5-24 / 3	5	4.2
30	6367	1	3-23	6-06 / 1	4	1.4
31	6368	7	3-23	6-13 / 2	3	3.5
32	6369	9	3-23	6-19 / 3	4	14.6
33	6299	1	3-48	3-09 / 2	4	6.3
34	6149	5	3-48	5-12 / 1	4	3.8
35	6304	1	3-48	7-27 / 1	2	9.8
36	6300	1	4-34	3-03 / 2	6	0.4
37	6148	1	4-34	4-17 / 3	4	4.6
38	6145	4	4-34	5-05 / 3	6	1.8
39	6137	29	4-39	3-10 / 3	6	3.8
40	6136	2	4-59	5-24 / 3	5	9.1
41	6132	3	5-51	4-17 / 2	6	1.7
42	6133	1	5-51	4-23 / 2	5	2.1
43	6357	1	5-51	5-24 / 3	5	1.7

Clones represent scFv with the same V(D)J recombination that were either identical, carried mutations in the VH gene or paired with a different VL gene compared to the prototype scFv. The table is sorted on V, D and J gene families consecutively. The average nucleotide mutation frequency of each VH gene is shown.

**Figure 3:**
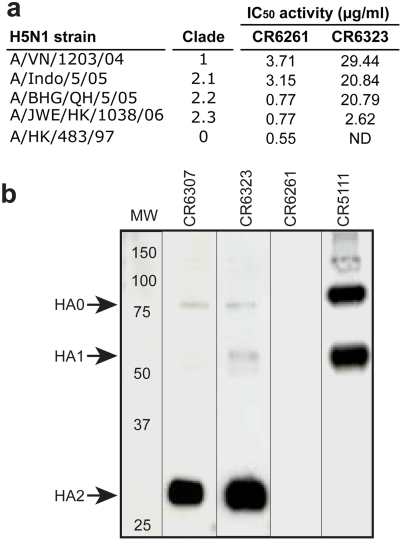
Cross-clade H5N1 neutralizing activity and HA subunit localization. (a) Neutralizing activity of purified IgG1 against 100 TCID_50_ of H5N1 viruses, the 50% neutralizing concentration (IC_50_) was calculated by the Spearman-Karber method. (b) Immunoblot of H5 rHA probed with indicated IgG1. Molecular weight marker (MW) and subunit locations indicated.

Next we converted all 91 distinct scFv into full length IgG1 and characterised them based on binding and neutralization activity. A group of 55 clones represented by 13 mAbs with a unique V(D)J recombination were characterised by strong binding to different H5 rHA antigenic formats (data not shown) and *in vitro* neutralizing activity (Table 2). The most potent neutralizing mAb was CR6261, which demonstrated broad neutralizing activity against a panel of antigenically diverse H5N1 viruses isolated over the last decade (Fig. 3a). The neutralizing potency range (0.6–3.7 µg/ml) of CR6261 is similar to previously reported H5N1 neutralizing monoclonals (0.07–7 µg/ml) isolated from immortalized B cells of H5N1 survivors [14]. However surprisingly, neither CR6261 nor the other neutralizing mAbs exhibited hemagglutination inhibition activity which is typically associated with neutralizing activity [30] (Table 2). Western blot analysis of the mAb panel partially explained this result by identifying two unique neutralizing mAbs (CR6307 and CR6323) that bound to the HA2 subunit of HA (Fig. 3b), a membrane proximal region spatially distinct from the receptor binding domain in HA1 responsible for hemagglutination [31]. Other non-neutralizing mAbs also showed binding to HA2 (data not shown) but only one non-neutralizing mAb (CR5111) was shown to bind the HA1 subunit (Fig. 3b; Table 2). Competition ELISA showed that 10/13 neutralizing mAbs, including CR6261, that were negative in the western blot analysis, completely blocked binding of labelled CR6323 to H5 rHA (Table 2) suggesting they recognise the same antigenic region in HA2. Conversely, CR6307 which was mapped to HA2 (Fig. 3b) only weakly inhibited CR6323 (Table 2).

**Table 2:** Characteristics of the neutralizing mAb panel.

IgG1	Clones	IGHV	Neutralizing activity NIBRG-14 (µg/ml)		HAI (µg/ml)	H5 competition ELISA % binding to HA		
			IC_100_	IC_50_	IC_50_	CR6261-FITC	CR6323-FITC	
CR6261	23	1-69	0.78	0.55	>50	6	2	
CR6329	1	1-69	3.1	1.9	>50	21	3	
CR6342	1	1-69	6.3	2.19	>50	12	9	
CR6325	2	1-69	6.3	2.6	>50	12	2	
CR6327	1	1-69	6.3	2.6	>50	24	12	
CR6323	18	1-69	6.3	3.7	>50	2	-6	
CR6332	1	1-69	13	4.5	>50	9	-5	
CR6334	1	1-69	6.3	4.5	>50	15	6	
CR6344	1	1-69	25	6.3	>50	17	8	
CR6307	1	3-21	25	8.9	>50	60	79	
CR6331	1	1-69	25	10	>50	5	-3	
CR6343	1	1-69	50	13	>50	52	56	
CR6262	3	1-69	25	15	>50	47	44	
CR5111	8	3-21	>100	>100	>50	86	113	
Neg. Ctrl	NA	1-69	>100	>100	>50	100	108	

Hemagglutination inhibition (HAI) and microneutralizing assay are described in the methods. Competition ELISA measures the % binding of labelled CR6261 or CR6323 to H5 rHA after pre-incubation with saturating concentrations of unlabelled IgG1.

### Identification of donor library yielding H5N1 neutralizing mAbs

**Figure 4:**
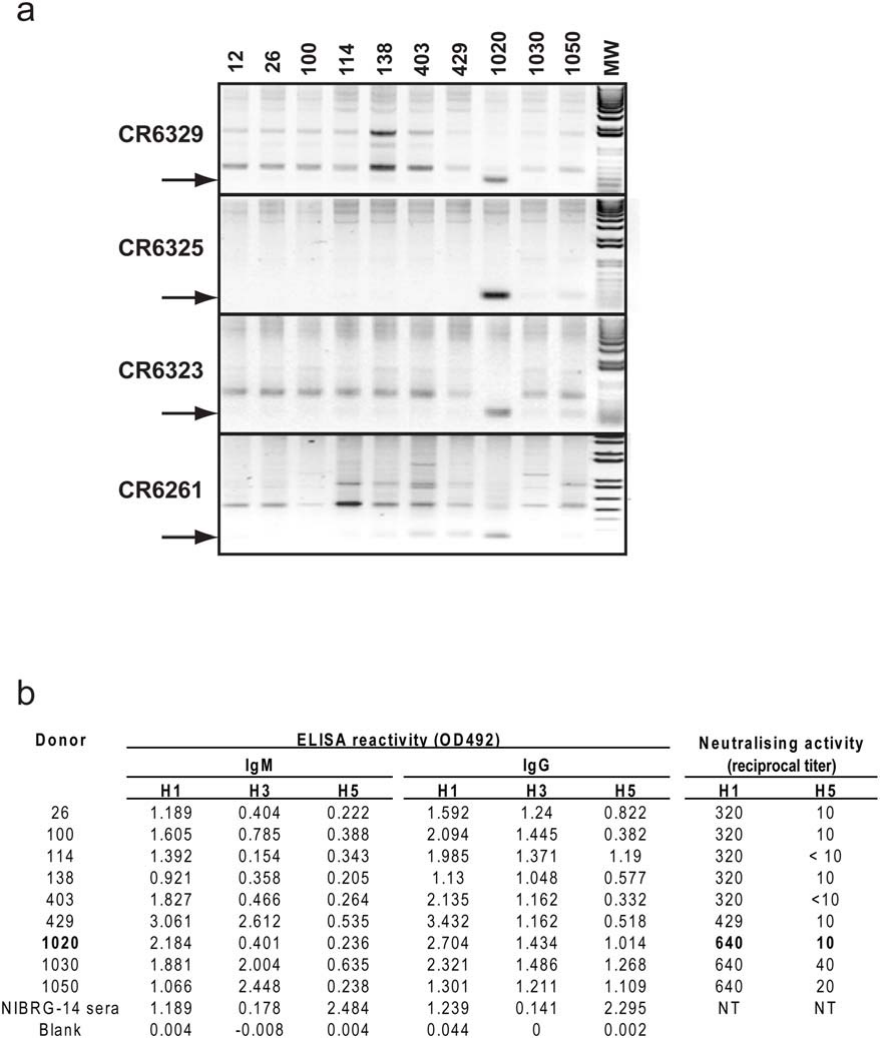
PCR screen of individual donor libraries for neutralizing mAbs and donor serology. (a) PCR amplification of cDNA from each donor IgM^+^ memory B cell library using oligonucleotide pairs designed so their 3′ ends specifically anneal in the HCDR1 and HCDR3 regions. Donors are indicated at the top of the figure. The expected size of the amplified fragment is indicated with an arrow. The identity of the bands was confirmed by sequencing (b) Binding and neutralizing activity of donor serum collected at the same time as the B cells used for library construction (note serum was not available for donor 12). IgM and IgG ELISA reactivity was measured against rHA and neutralizing activity against H1N1 (A/Hong Kong/54/98) and H5N1 (A/Vietnam/1203/04). Donor 1020 who was PCR positive for the tested neutralizing mAbs is indicated in bold.

Because the IgM^+^ memory B cell libraries were pooled before selection it was not possible to immediately determine if the neutralizing mAbs were rescued from B cells of a single donor or different donors; we addressed this issue in two ways. Analysis of individual IgM^+^ memory B cell libraries by specific RT-PCR and sequencing showed one of the three seasonal influenza vaccinated donors (#1020) was the source of at least 4 of the neutralizing mAbs (Fig. 4a). This was confirmed by the isolation of scFv with sequences identical to CR6261 in repeated selections with the single library constructed from this donor (data not shown). Remarkably, serum anti-H5 binding and H5N1 neutralizing titres for donor #1020 were no different to the other vaccinated or the unvaccinated donors (Fig. 4b) indicating that if H1N1/H5N1 cross-reactive IgM^+^ memory B cells were present in donor #1020 at the time of vaccination they did not mount a conventional amnestic response and represent a small fraction of the overall H1N1 response.

### Breadth of heterosubtypic influenza virus neutralization by the H5N1 human mAb panel

**Figure 5:**
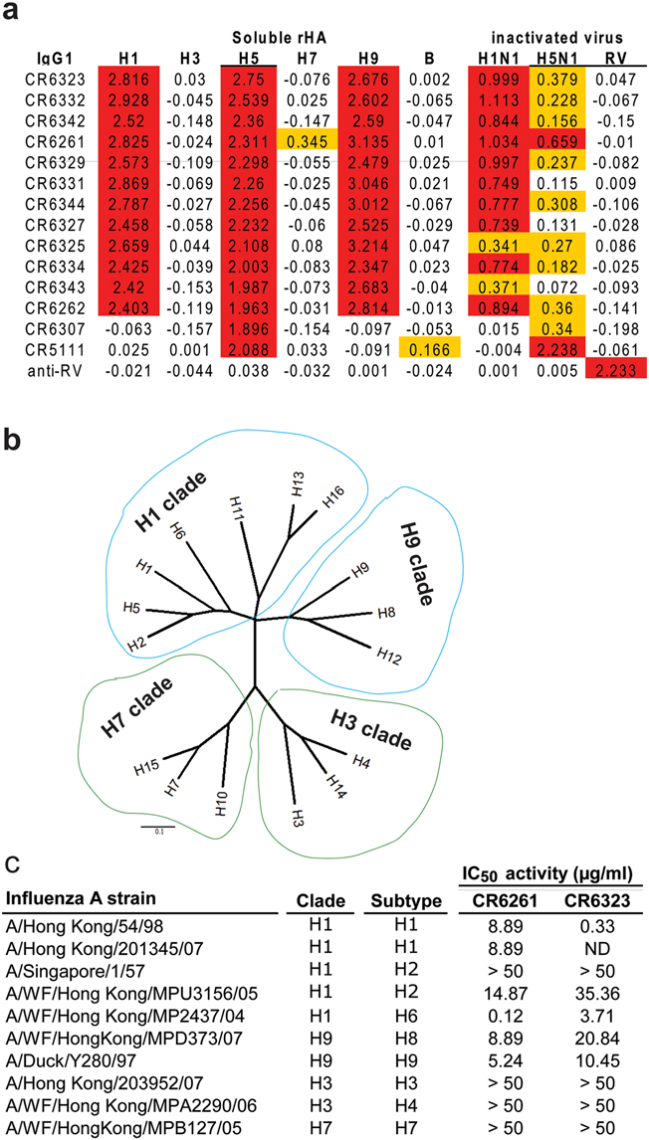
Heterosubtypic binding and neutralizing activity. (a) The binding activity of the IgG1 panel (5 µg/ml) was measured against 0.5 µg/ml directly coated recombinant HA antigen (see methods for strain designation), BPL inactivated NIBRG-14 (HA from A/Vietnam/1194/04) or BPL inactivated H1N1 (A/New Caledonia/1/99). Detection was performed with mouse anti-human IgG-HRP and results given as OD_492_ nm. Control antigens were Influenza B virus (B/Ohio/1/05) and Rabies vaccine (RIVM, Bilthoven). Anti-rabies virus (RV) IgG1 (IGHV1-69) was used as a negative control. ELISA values>10× background or between 10× and 3× background are coloured red and yellow respectively. (b) Phylogenetic tree of amino acid sequences at the subtype level, division of subtypes by group is indicated by coloured balloons (blue H1 and H9, green H3 and H7). (c) Neutralizing activity of purified IgG1 against 100 TCID_50_ of influenza A viruses, IC_50_ calculated as in Fig. 3.

Given the conserved nature of the HA2 subunit and its known ability to induce cross-reactive immune responses [32] we examined whether the H5N1 neutralizing mAbs in the panel had heterosubtypic activity. In solid phase ELISA all mAbs recognising the antigenic region of CR6323 and CR6261 demonstrated equivalent strong reactivity to rH1, rH5 and rH9 but not rH3, rH7 or rHA from a B virus (Fig. 5a). To exclude the possibility that the antibodies cross-reactive properties extended to autoreactivity as has been described for several broadly neutralizing HIV mAbs [33] they were screened by immunohistochemistry on a panel of 32 human tissues. No evidence of strong specific immunoreactivity was observed in any tissue when compared with isotype matched control antibody (data not shown).

The monovalent affinity of CR6261, CR6323 and CR6307 was measured by Biacore. The affinity of CR6261 and CR6323 was similar for rH1, rH5 and rH9 HA and in the low nanomolar range (Table 3), however while the affinity of CR6307 is high for rH5 it recognises a different antigenic region in HA2 that is not conserved in other influenza subtypes (Fig. 5a). To determine the full breadth of CR6261 and CR6323 heterosubtypic neutralizing activity, representative strains from 9 subtypes were compared. Phylogenetic analysis based on published methodology [34] demonstrates a subdivision of HA's, consistent with previous reports, that falls into two main groups [35] and four clades [36]: the H1 and H9 clade in one group and the H3 and H7 clade in the other group (Fig. 5b). CR6261 neutralized all H1 clade and H9 clade viruses, except for one of the H2N2 isolates, and potency among subtypes was relatively well conserved (Fig. 5c) consistent with affinity measurements (Table 3). CR6323 also neutralised the same strains as CR6261 but 10 fold variability in subtype neutralizing activity was measured that contrasts with its equivalent affinity for H1, H5 and H9 subtypes (Fig. 5c). CR6323 binds a different epitope than CR6261 (Fig. 3b) but in the same antigenic region (Table 2) suggesting that subtype differences in the presentation of this epitope impact on functional activity. Consistent with binding data, none of the tested strains from the H3 or H7 clade were neutralized by the mAbs (Fig. 5c) including CR6261 for which weak but consistent ELISA reactivity was measured against rH7 HA. Based on this analysis the antigenic region recognized by CR6261 and CR6323 appears to segregate with a previously characterized division of HA's that contains, H1, H2, H5, H6, H8, H9, H11, H12, H13 and H16 subtypes [36].

**Table 3:** Monovalent affinity of Fabs measured by surface plasmon resonance against rHA.

mAb	HA	Ka (1/Ms)	Kd (1/s)	KD (M)
CR6261	H1	9.8×10^5^	3.7×10^−3^	3.8×10^−9^
	H5	2.7×10^5^	1.1×10^−3^	4.1×10^−9^
	H9	6.8×10^5^	3.7×10^−3^	5.4×10^−9^
CR6323	H1	1.4×10^5^	5.4×10^−4^	3.9×10^−9^
	H5	1.9×10^5^	1.2×10^−3^	6.3×10^−9^
	H9	1.3×10^6^	2.7×10^−3^	2.1×10^−9^
CR6307	H5	7.0×10^4^	4.5×10^−4^	6.4×10^−9^

### Identification of the HA binding region for the heterosubtypic neutralizing mAbs

**Figure 6:**
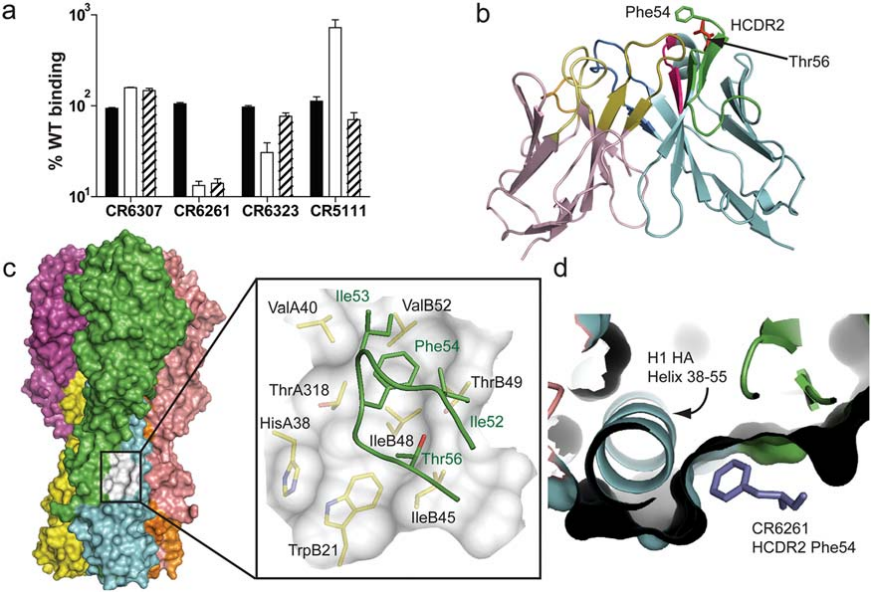
Identification of the antigenic region of neutralizing mAbs. (a) FACS binding of IgG1 to surface expressed H5 rHA was measured after sequential treatment with trypsin (solid bars), pH 4.9 buffered medium (open bars) and DTT (striped bars) and expressed as percentage binding to untreated rHA from two independent experiments (mean±s.e.m.). (b) Global view of CR6261, VL is rose, VH is blue, HCDR2 is green, Thr56 red and Phe54 green. (c) Surface representation of trimeric H1 HA (A/South Carolina/1/18; 1RUZ). HA1 subunits are green, burgundy and pink, HA2 subunits yellow, blue and orange. The hydrophobic pocket is white and shown magnified as an inset with the HCDR2 of CR6261 in green docked to the structure. HA residues around and forming the hydrophobic pocket are labelled in black and HCDR2 residues in the pocket are green. (d) perpendicular view of the hydrophobic pocket down the axis of helix 38–55 occupied by Phe54 of HCDR2 in purple.

Conserved structural correlates have been identified that support the classification of HA subtypes into groups and clades as described above [37]. These structural features are predominantly located in the more conserved HA2 fusion subdomain. This region undergoes a dramatic structural rearrangement between the neutral (native) and low pH (fusion) forms of HA [38]. Not surprisingly binding of CR6261 to surface exposed H5 rHA was lost when subjected to low pH treatment, yet remarkably the epitopes of both CR6323 and CR6307 were conserved under these conditions (Fig. 6a). The only significant solvent exposed region in the HA2 subunit not rearranged during this conformational shift is the helix 38–55 (region A) [38] which may form part of the antigenic region recognized by CR6307 and the cross-neutralizing mAbs (CR6323 and CR6261).

**Figure 7:**
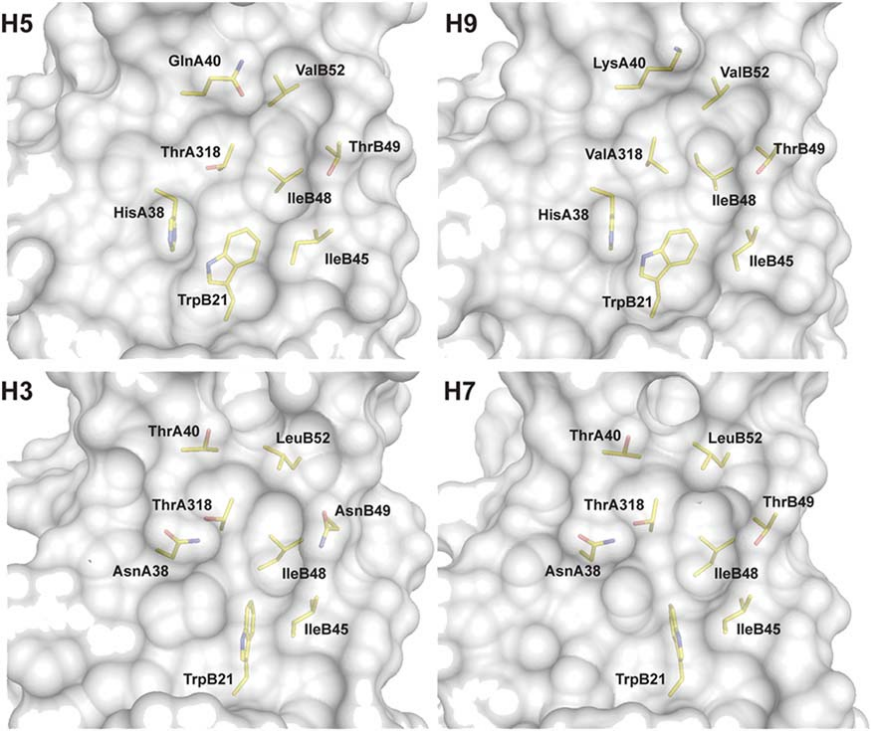
The hydrophobic pocket region in the stem domain of different HA subtypes. Surface representation of the hydrophobic pocket recognized by CR6261 from crystal structures of H5 (2IBX) and H9 (1JSD) and the corresponding region from crystal structures of H3 (1MQM) and H7 (1TI8). HA residues around and forming the hydrophobic pocket are indicated in black, H3 numbering is used throughout for consistency. Note the H38N and Q40T replacements in the H3 and H7 structures that introduce a potential glycosylation site in the region.

We next investigated the interaction of CR6261 with the helix 38–55 region by modelling. A 3-D structure of the VH-VL region of CR6261 was modelled by homology to framework regions and CDR fragments from existing x-ray crystal structures (Fig. 6b). A striking feature of the subtype cross-reactive mAbs was their restriction to the VH gene IGHV1-69 (Table 2) which exposes conserved hydrophobic residues at the tip of the HCDR2 loop, in particular a phenylalanine at position 54 (Fig. 2; Fig. 6b). Selective IGHV1-69 germline restriction has been observed for mAbs binding defined epitopes of gp120 [39] and gp41 [40] from HIV-1 virus and the analysis of the co-crystal of one, D5 and its target an α-helix in the gp41 inner-core trimer (2CMR), has shown that these hydrophobic residues are required to stabilize binding [40]. Using the structure 2CMR as a guide, the CR6261 model was docked in the region of helix 38–55. From this initial position a concave hydrophobic patch formed by the residues Ile45, Ile48, Thr49, Val52 and Ile56 of HA2 and His38 and Val40 of HA1 (H3 numbering throughout paper) was identified that could accommodate the hydrophobic residues of HCDR2 (Fig. 6c) and in particular Phe54 (Fig. 6d). Consistent with the binding and neutralizing properties of CR6261 and the structural classification of HA subtypes [37] the identified hydrophobic patch is conserved in x-ray crystal structures of the H1 and H9 group but not in the H3 and H7 group (Fig. 7).

### Confirmation of the stem domain hydrophobic pocket as the binding region of the heterosubtypic neutralizing mAbs

**Figure 8:**
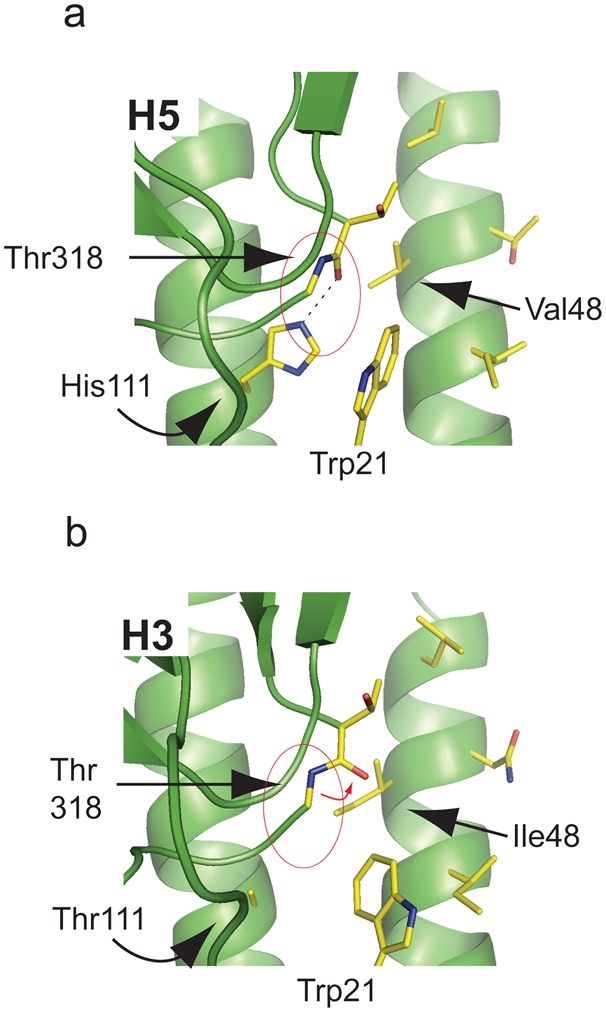
Generation of CR6261 neutralisation escape variants. (a) The hydrogen bond interaction of HA2 His111 with HA1 Thr318 is shown (circled red) in the H5 structure 2IBX. (b) An H3 structure (1MQM) is shown where the peptidic plane re-orientation of Thr318 is indicated by a red arrow.

Several pieces of experimental evidence support the localisation of the antigenic region. First, CR6261 neutralisation escape variants of RG-A/Indonesia/5/05 were generated, after extensive passaging, that harboured a fixed mutation (H111L) in the HA2 subunit; viruses with this unique mutation were confirmed to be resistant to CR6261 neutralisation (IC_50_>100 µg/ml vs. 6.25 µg/ml for wild type). His111 is a conserved H1 and H9 group specific residue [37] but is buried in the prefusion state and so not accessible to direct antibody interaction. Comparison of an H5 structure (Fig. 8a) to an H3 structure where threorine is in position 111 of HA2 (Fig. 8b) shows that when hydrogen bonding is lost between the His111 side chain and the Thr318 main chain (Fig. 8a,b; circle) as would occur with the H111L escape mutation (because Leu111 like Thr111 cannot form a hydrogen bond with Thr318), the peptidic plane of Thr318 is re-orientated introducing an exposed carbonyl moiety into the binding pocket (Fig. 8b; arrow). We predict that the hydration of the carbonyl will modify the hydrophobic character of the pocket explaining the loss of CR6261 neutralizing activity.

**Figure 9:**
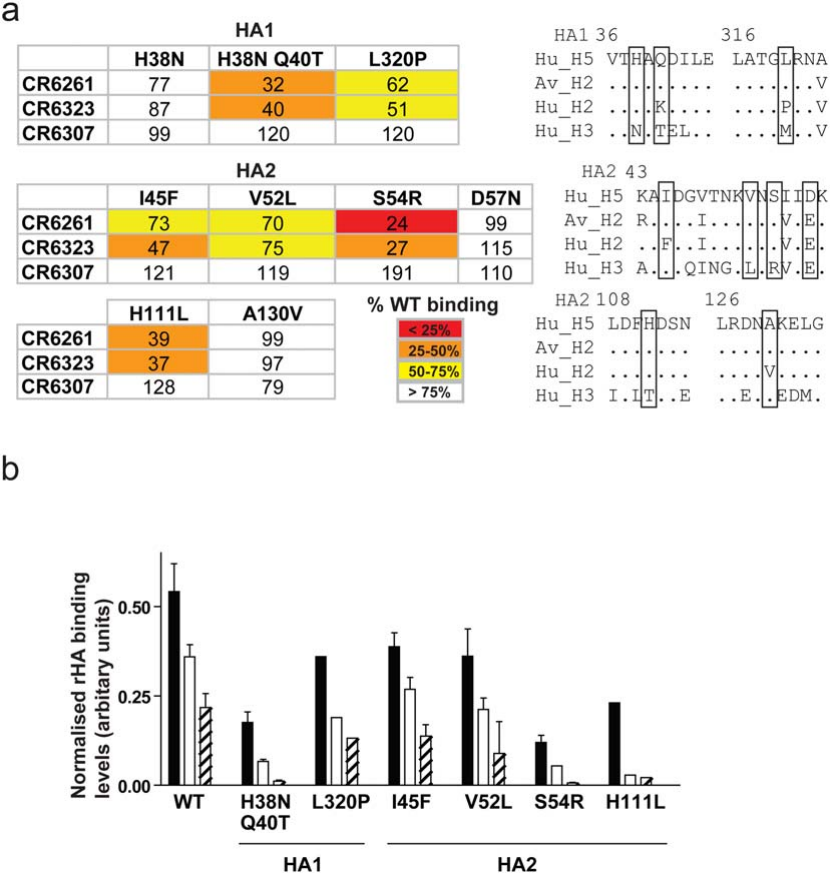
Binding of CR6261 to H5 HA point mutants. (a) FACS binding of IgG1 to surface expressed mutants of H5 rHA. Mean fluorescent intensity (MFI) values were normalised by NIBRG-14 antiserum reactivity to the corresponding mutant and expressed as the average percentage of wild type H5 rHA binding from two independent experiments (see key for colour code). Adjacent are sequence alignments of the corresponding region, mutated residues are boxed. (b) FACS binding activity to WT or mutant H5 rHA performed as above for CR6261 (solid bars), CR6261 with the HCDR2 mutation F54L (open bars) or F54A (striped bars). MFI was normalised as above and expressed as mean±s.e.m. arbitrary units from two independent experiments.

Second, when H5 residues were substituted for corresponding H3 residues in the hydrophobic pocket reductions in surface expressed H5 rHA binding were measured for CR6261 and CR6323 (Fig. 9a). This was particularly true of the double mutation H38N and Q40T (HA1) which introduces a glycosylation site in the pocket. Binding reductions were also seen for the two H5 to H2 mutants, L320P (HA1) and I45F (HA2), which introduced residues in the hydrophobic pocket unique to the non-neutralised human H2N2 strain (Fig. S1) but not for the H5 to H2 mutation A130V (HA2) which is outside the antigenic region; similarly the H5 to H3 mutation D57N (HA2) which is adjacent to the hydrophobic pocket did not affect binding (Fig. 9a). None of the mutations introduced into the hydrophobic pocket completely blocked binding of CR6261 or CR6323, however the 60% binding reduction measured for the neutralisation escape mutation H111L (HA2) suggests that incomplete blocking could still be functionally relevant (Fig. 9a). This may reflect on one hand the additive effect of the residues forming the hydrophobic pocket such that a single substitution does not destroy interaction and on the other hand the contribution provided by HCDR3 and the other CDR loops. In this regard the HA2 mutation S54R is outside the hydrophobic pocket but has a substantial impact on binding (Fig. 9a).

Although our data argue that the IGHV1-69 CDR2 of CR6261 and the other mAbs has a critical role in forming the antigen binding site, this VH gene does not confer HA specificity by itself. A VH gene matched control antibody binding rabies virus glycoprotein failed to bind or neutralise H5N1 viruses (Table 2) and no H5 rHA reactivity was measured with a panel of eight West Nile virus envelope specific mAbs that use the IGHV1-69 gene (data not shown). Rather the proposed role of the hydrophobic residues on the tip of the HCDR2 loop is in anchoring HCDR3 and other CDR loops and perhaps orientating their spatial interaction [39]. Consistent with this, substitution of Phe54 at the tip of the HCDR2 loop of CR6261 with leucine or alanine reduced binding in relation to hydrophobicity, and additive decreases in binding were observed when CR6261 mutants and HA mutants were combined (Fig. 9b).

Finally we observed that CR6307, which does not use the IGHV1-69 germline gene, is not subtype cross-reactive and only weakly competes with CR6261 or CR6323 for binding to rHA, does not appear to be sensitive to the mutations around the hydrophobic pocket (Fig. 9a), however the mutation S54R influences its binding suggesting like the fusion shift data (Fig. 6a) that its epitope is also located in the 38–55 helix region.

### In vivo activity of heterosubtypic neutralizing mAb CR6261 against H5N1 and H1N1 viruses

**Figure 10:**
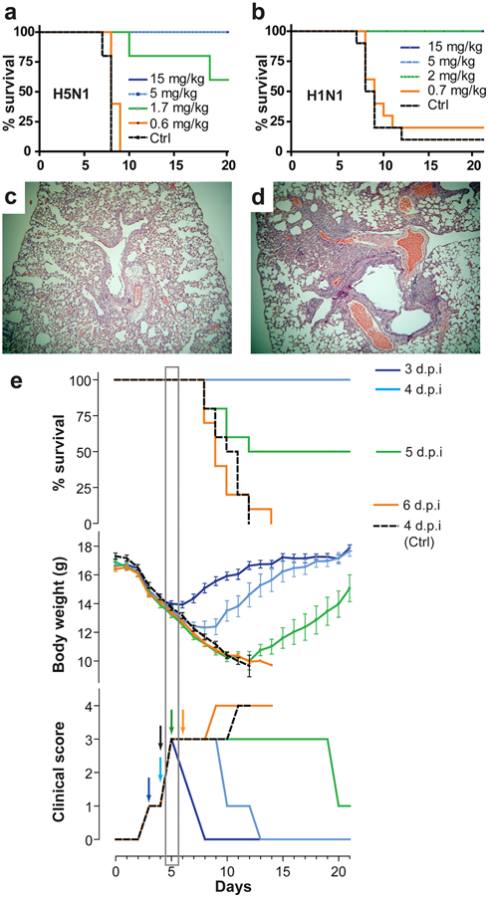
In vivo protective activity of CR6261 against wild type H5N1 and H1N1 strains. Kaplan-Meier survival curves of BALB/c mice were injected (i.p.) with CR6261 or irrelevant control (15 mg/kg) then challenged 24 h later (i.n.) with (a) 10 LD_50_ of A/Vietnam/1203/04 (n = 5) or (b) 25 LD_50_ A/WSN/33 (n = 10) and observed daily for a period of 21 days. Haematoxylin-Eosin stained lung sections taken 6 d.p.i from (c) CR6261 or (d) control mAb treated mice (1 d.p.i.) and challenged with 25 LD_50_ A/Hong Kong/156/97. (e) Survival (upper panel), mean±s.e.m. body weight, (middle panel) and median clinical signs (bottom panel) of mice (n = 10) challenged as in (c,d) and injected i.v. with 15 mg/kg CR6261 IgG1 3 (dark blue), 4 (light blue), 5 (green) or 6 days (orange) or control mAb (black dotted line) 4 days after challenge (see colored arrows in bottom panel). The day five time point at which therapeutic efficacy is lost is indicated by a grey box.

We examined the *in vivo* potency of CR6261 in murine lethal challenge models. Prophylactic administration of CR6261 (5 mg/kg; i.p.) was 100% protective in a dose titration against 10 LD_50_ A/Vietnam/1194/04; none of the CR6261 treated mice showed signs of infection, in contrast all mice that received isotype control mAb (15 mg/kg; i.p.) suffered from respiratory symptoms by day 4 and succumbed to infection or were sacrificed by day 8 (Fig. 10a). Similarly, CR6261 fully protected mice against 25 LD_50_ of the mouse adapted H1N1 strain A/WSN/33 at 2 mg/kg (Fig. 10b) thus demonstrating cross-protection. Administration of CR6261 (15 mg/kg; i.p.) to mice (n = 10) one day after infection (i.n.) with 25 LD_50_ of A/Hong Kong/156/97 or A/WSN/33 fully protected all animals. Lung pathology in H5N1 infected mice (n = 3) six days post-infection showed signs only of mild lymphohistiocytic cell extravasation in CR6261 treated mice (Fig. 10c) while evidence of bronchiolitis and moderate to severe pneumonia was recorded in all controls (Fig. 10d).

Clinical interventions to treat H5N1 infections generally begin after the onset of symptoms. To establish the effective treatment window of CR6261 a single dose (15 mg/kg) was injected i.v. to rapidly achieve peak circulating concentrations, 3, 4, 5 or 6 days after challenge with 25 LD_50_ A/Hong Kong/156/97 (Fig. 10e). A rapid reversal of mean weight loss and clinical signs of infection was measured when CR6261 was given three days or four days post challenge, time points at which mice had lost 10% and 15% of body weight respectively; all mice in these treatment groups regained their full body weight by the end of the observation period. Remarkably, CR6261 treatment at day five post-infection, when all mice displayed signs of respiratory distress, was still able to protect 50% of mice (Fig. 10e, grey box). Our observations with CR6261 in murine lethal challenge models extend the findings of others [14], [41] and are consistent with the proposal that intervention with influenza virus neutralizing mAbs may have clinical utility in human H5N1 cases.

## Discussion

In this study we have built combinatorial libraries and selected a panel of human monoclonal antibodies against H5 HA from a particular compartment of memory B cells that are characterized by their continued expression of IgM on the surface. Our results suggest that IgM^+^ memory B cells express mAbs recognizing T-dependent antigens at high affinity, and consistent with a hallmark of memory B cells, all selected H5N1 mAbs contained mutated VH genes. Memory B cells are also generally defined by their rapid proliferation and differentiation into antibody secreting plasma cells on re-exposure to antigen. Yet from this study it is not clear that the IgM^+^ memory B cells expressing the cross-neutralizing HA specificities participate in a true recall response upon vaccination. An increase in serum neutralizing activity was measured in all vaccinated donors for H1N1 but not H5N1, even in donor #1020 from which the heterosubtypic neutralizing mAbs were isolated. Thus if the B cell clones expressing CR6261 and the other cross-reactive mAbs were present in the donor prior to vaccination (as would be expected) then a true recall response associated with differentiation into plasma cells has not likely occurred. Although we can not exclude the possibility that at later time points serological differences could have been measured the results are consistent with the observation that measurable heterosubtypic immunity is rare in the general population [10].

We have not made a comparative analysis of other B cell compartments to investigate whether related clones are present in isotype switched memory cells or plasma cells of donor #1020 or other donors. However, preliminary data for a mAb isolated from the bone marrow of an H5N1 infected patient was recently reported that possesses cross-neutralizing activity between H5N1 and H1N1 [13]. Remarkably this mAb uses the IGHV1-69 germline gene and conserves the hydrophobic residues at the tip of the HCDR2 loop that we show in this study to be critical in binding the conserved hydrophobic pocket of HA. Unfortunately without further data it can only be inferred that this mAb is binding to the same region as CR6261 and other mAbs described in this study. The ease at which the IgM^+^ memory B cell repertoire can be accessed in normal, infected and vaccinated donors compared to bone marrow and the diversity in this repertoire compared to antibody secreting cells [16] supports this approach to generate therapeutic mAbs against other pathogenic targets and should be further explored.

The epitopes of CR6261, CR6323 and the other cross-neutralizing mAbs were localised to the stem domain of HA using a combination of homology modelling, mutagenesis and mapping with escape variants. This region is highly conserved and, based on comparisons at a sequence and structural level, appears to segregate into two groups one containing H1, H2, H5, H6, H8, H9, H11, H12, H13 and H16 subtypes and the other H3, H4, H7, H10, H14 and H15 subtypes. The neutralizing mAbs described in this study recognize antigenic determinants in the first group that confer different levels of neutralization potency against these subtypes but do not recognise representative virus strains from subtypes in the second group tested so far. Escape mutants of a murine anti-H2N2 mAb (C179) with cross-neutralizing activity to H1 subtypes but not H3 subtypes have also placed its epitope in this region [42]. C179 has relatively weak neutralizing and protective activity against H1 and H5 viruses (e.g. protection from a lab adapted LPAI H5N2 strain required 70 mg/kg of C179) [43], [44]. Preliminary evidence suggests C179's mechanism of action is related to fusion inhibition rather than blocking of attachment [44]. Although we have not examined the neutralisation mechanism of the human mAbs identified in this study they, like C179, do not have hemagglutination inhibition (HAI) activity. Importantly, we show that mAbs like CR6261 targeting this region can have potent prophylactic and therapeutic activity and that humans are capable of generating antibodies directed to this region. Vaccines with broad heterosubtypic protective activity are being actively pursued, particularly against matrix protein 2 (M2) [45]–[47], however many uncertainties remain about their effectiveness [10]. Vaccination studies have been performed with the HA2 subunit but have not demonstrated robust protective responses in experimental models [32], [48]. The information provided here on the conserved antigenic site in the HA stem domain could provide a new avenue for targeted vaccine strategies.

Development of clinical interventions against a potential influenza pandemic are challenged by uncertainty over future pandemic viral subtypes [1], [3], [5], [49], antigenic variability within subtypes and the potential for antiviral resistance to emerge [7], [8], as recently observed in H1N1 strains [9]. The mAb CR6261 identified in this study potentially addresses these issues. First, it neutralises most of the virus subtypes that currently show pandemic potential (i.e. H2, H5, H6 and H9 viruses). Second, it was demonstrated to neutralise all five H5N1 virus clades and subclades tested, which represent viruses isolated from 1997 to 2006. The generation of CR6261 neutralisation escape variants required extensive viral passaging suggesting that alteration of its epitope is not easily accomplished without negatively influencing virus infectivity. The prophylactic and therapeutic utility of CR6261 against different H5N1 strains and influenza A subtypes argue for its development as an important alternative or adjunct to currently stockpiled pandemic interventions.

## Supporting Information

Figure S1 Alignment of H2N2 HA sequences. (a) Amino acid alignment of sequences from CR6261 and CR6323 neutralised and non-neutralised influenza strains. Only residues that differ from the top sequence Human H2N2 (Hu_H2; A/Singapore/1/57) are shown, H3 numbering is used. Other sequences were Avian H2 (Av_H2; A/WF/Hong Kong/MPU3156/05), Human H5N1 (Hu_H5; A/Vietnam/1203/04), Avian H5N1 (Av_H5; A/Japanese white eye/Hong Kong/1038/06), Human H1N1 (Hu_H1; A/Hong Kong/54/98) and Avian H9N2 (Av_H9; A/Duck/Y280/97). Antigenic sites based on H1 are boxed Ca1 (red), Ca2 (burgundy), Cb (green), Sa (light blue) and Sb (yellow). Human H2N2 residues that fall outside the predicted antigenic sites and differ from the CR6261 neutralised avian H2N2 strain are boxed on the top sequence. (b) the antigenic sites from above are shown mapped onto a surface representation of H1N1 crystal structure (1RUZ) using the colour scheme above, the boxed unique residues of the non-neutralised human H2N2 strain are marked in dark blue on the surface.
(0.08 MB DOC)
