# Supplementary figures and images for: Heterosubtypic Neutralizing Monoclonal Antibodies Cross-Protective against H5N1 and H1N1 Recovered from Human IgM+ Memory B Cells
Source: PLoS One. 2008 Dec 16;3(12):e3942. doi: 10.1371/journal.pone.0003942 (PMC2596486; doi:10.1371/journal.pone.0003942)

a

**a**

b

**b**


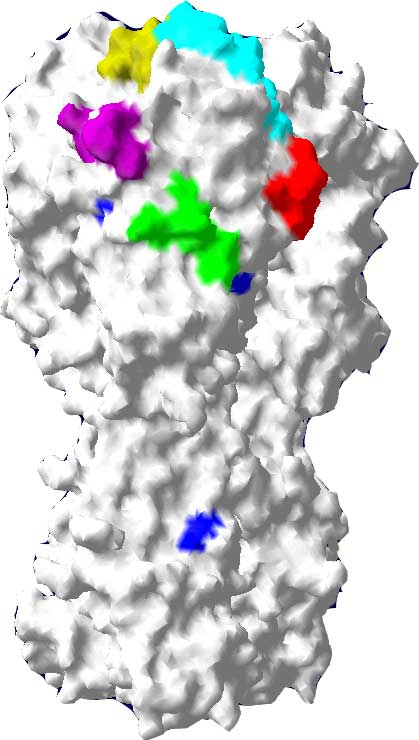


**S114K (HA1)**

**N96D (HA1)**

**I45F (HA2)**

Supplement: Figure S1 — Alignment of H2N2 HA sequences. (a) Amino acid alignment of sequences from CR6261 and CR6323 neutralised and non-neutralised influenza strains. Only residues that differ from the top sequence Human H2N2 (Hu_H2; A/Singapore/1/57) are shown, H3 numbering is used. Other sequences were Avian H2 (Av_H2; A/WF/Hong Kong/MPU3156/05), Human H5N1 (Hu_H5; A/Vietnam/1203/04), Avian H5N1 (Av_H5; A/Japanese white eye/Hong Kong/1038/06), Human H1N1 (Hu_H1; A/Hong Kong/54/98) and Avian H9N2 (Av_H9; A/Duck/Y280/97). Antigenic sites based on H1 are boxed Ca1 (red), Ca2 (burgundy), Cb (green), Sa (light blue) and Sb (yellow). Human H2N2 residues that fall outside the predicted antigenic sites and differ from the CR6261 neutralised avian H2N2 strain are boxed on the top sequence. (b) the antigenic sites from above are shown mapped onto a surface representation of H1N1 crystal structure (1RUZ) using the colour scheme above, the boxed unique residues of the non-neutralised human H2N2 strain are marked in dark blue on the surface. (0.08 MB DOC) [file pone.0003942.s001.doc]
